# Temporal pattern of questing tick *Ixodes ricinus* density at differing elevations in the coastal region of western Norway

**DOI:** 10.1186/1756-3305-7-179

**Published:** 2014-04-11

**Authors:** Lars Qviller, Lise Grøva, Hildegunn Viljugrein, Ingeborg Klingen, Atle Mysterud

**Affiliations:** 1 Centre for Ecological and Evolutionary Synthesis (CEES), Department of Biosciences, University of Oslo, P.O. Box 1066, Blindern NO-0316 Oslo, Norway; 2 Bioforsk - Norwegian Institute for Agricultural and Environmental Research, Organic food and farming Division, NO-6630 Tingvoll, Norway; 3 Norwegian Veterinary Institute, P.O. Box 750, Sentrum NO-0106 Oslo, Norway; 4 Bioforsk - Norwegian Institute for Agricultural and Environmental Research, Plant Health and Plant Protection Division, Fr.A. Dahlsvei 20, NO-1430 Ås, Norway

**Keywords:** *Ixodes ricinus*, Ticks, Tick-borne diseases, Climate change, Prevailing weather, Tick phenology, Temperature, Desiccation stress

## Abstract

**Background:**

Climate change can affect the activity and distribution of species, including pathogens and parasites. The densities and distribution range of the sheep tick (*Ixodes ricinus*) and it’s transmitted pathogens appears to be increasing. Thus, a better understanding of questing tick densities in relation to climate and weather conditions is urgently needed. The aim of this study was to test predictions regarding the temporal pattern of questing tick densities at two different elevations in Norway. We predict that questing tick densities will decrease with increasing elevations and increase with increasing temperatures, but predict that humidity levels will rarely affect ticks in this northern, coastal climate with high humidity.

**Methods:**

We described the temporal pattern of questing tick densities at ~100 and ~400 m a.s.l. along twelve transects in the coastal region of Norway. We used the cloth lure method at 14-day intervals during the snow-free season to count ticks in two consecutive years in 20 m^2^ plots. We linked the temporal pattern of questing tick densities to local measurements of the prevailing weather.

**Results:**

The questing tick densities were much higher and the season was longer at ~100 compared to at ~400 m a.s.l. There was a prominent spring peak in both years and a smaller autumn peak in one year at ~100 m a.s.l.; but no marked peak at ~400 m a.s.l. Tick densities correlated positively with temperature, from low densities <5°C, then increasing and levelling off >15-17°C. We found no evidence for reduced questing densities during the driest conditions measured.

**Conclusions:**

Tick questing densities differed even locally linked to elevation (on the same hillside, a few kilometers apart). The tick densities were strongly hampered by low temperatures that limited the duration of the questing seasons, whereas the humidity appeared not to be a limiting factor under the humid conditions at our study site. We expect rising global temperatures to increase tick densities and lead to a transition from a short questing season with low densities in the current cold and sub-optimal tick habitats, to longer questing seasons with overall higher densities and a marked spring peak.

## Background

The current global warming is changing biological activities and species distributions, and many species are experiencing a northward shift and an earlier onset of the seasonal timing of activity, termed phenology [1-3]. Of particular concern with rising temperatures and changes in precipitation patterns are changes in the distribution and range shifts in undesired organisms, such as parasites and disease agents [4]. In particular, the effects of climate change on vector-borne diseases may be difficult to predict because of the complexity of their transmission systems [5,6]. The changes in such systems are important for both animal and human welfare because of the zoonotic nature of many vector-borne diseases, including malaria [7], Lyme disease [8], and tick-borne encephalitis [9,10]. Among Europe’s emerging infectious diseases, both Lyme disease and tick-borne encephalitis are regarded as highly sensitive to warming effects [9,11]. Therefore, it is important to understand how the vector is affected by the prevailing weather patterns in different climatic regions.

The most important zoonotic disease-transmitting arthropod parasite in Europe is the sheep tick (*Ixodes ricinus*) [10]. Ixodid ticks are known to transmit pathogens to humans and other vertebrate hosts, causing Lyme disease, tick-borne encephalitis (TBE), anaplasmosis [12-14], and babesiosis [15]. *I. ricinus* populations currently appear to be increasing and expanding northwards and into higher elevations both in Scandinavia [11,16-18] and other parts of Europe (e.g., [19], in the UK and [20,21] in the Czech Republic). One suggested mechanism behind the reported increased distribution of ticks and tick-borne diseases is the rising temperatures linked to climate change, though changes in land use and growing host populations may also play a role [16,18,22].

The sheep tick is a three-stage, three-host tick that only attaches to a single host for a few days of its active life stage as it engorges [23]. The active life stage lasts approximately one year, and sheep ticks typically require 2–5 years to fulfil their life cycle. Most of their life is spent off host exposed to the prevailing weather [10,24,25]. The off-host periods are spent in developmental- or temperature-dependent diapause or questing for hosts [26]. Questing is the activity where the tick climbs up vegetation, extends its first pair of legs and waits for passing hosts. The onset of questing activity occurs when the daily maximum temperature reaches above 7°C for nymphs and adults and 10°C for larvae [26], but this may vary between regions [27]. Both temperature and vapour pressure deficit (VPD), a measure of the drying quality of air, affect tick abundance and activity patterns. Ticks are sensitive to desiccation [28]. For instance, a VPD above 4.4 mm Hg (equal to a relative humidity of 80% at 24°C) was shown to cause ticks to decent or stop their questing activity [29]. Ticks therefore divide their time between rehydrating in the litter layer and questing higher up [30]. To maintain their water balance, ticks actively take in water through a hygroscopic fluid that is produced in the salivary glands. This process costs energy, and maintaining this water balance is most likely the greatest constraint to tick activity [31]. Questing time is normally short: approximately 30% of the day for adult ticks [32]. Ticks remain in the litter layer, where the humidity is high, for the remainder of the time [33]. They can be active for days in favourable conditions, and descend from questing more often when VPD increases [29]. Tick questing activity has been linked to these climatic factors and is discussed throughout the literature [25,29-31,34-40].

The link between questing and prevailing weather implies that there may also be considerable variation between years in the actual pattern of questing. In dry regions, a tick’s seasonal questing activity often shows a bimodal tendency, with spring and autumn peak and with reduced midsummer activity. This is often considered to be because of the drought that follows high temperatures during midsummer [25,32,35], and this pattern is thus not expected in more humid, northern areas. TBE depends on co-feeding larval and nymphal ticks, and summer temperatures may affect the synchrony between tick instar stages [41,42]. Therefore, tick phenology and density linked to climate is an aspect of interest to further understand the recent increases in tick-borne diseases. Tick phenology is clearly also affected by life cycle considerations related to timing of egg laying, hatching of the different tick stages and induction of diapause, leading to fluctuations in the total tick population [43]. The number of questing ticks is also affected by moulting and the proportion of the population having found a host [43].

Studying the temporal pattern of tick questing densities along climate gradients may yield insight into future distributions under changing climatic conditions [36]. Recent studies report a strong negative effect of *I. ricinus* abundance with increasing elevation [36,44] and distance from the coast [44]. In the present study, we compared the temporal pattern of tick questing densities at two different elevations in two areas in Møre og Romsdal county on the western coast of Norway. We aimed to reveal possible differences in peak and onset of questing in two different climatic regimes in the same region. We did so by comparing questing tick densities at ~400 m a.s.l. (referred to as high elevations) and ~100 m a.s.l. (referred to as low elevations). Further, we aimed to link these differences in seasonal questing density patterns to local temperature and humidity (recorded by locally placed climate loggers).

## Methods

### Study area

Data were collected along transects at two localities in Møre og Romsdal county on the western coast of Norway: Tingvoll (62°54′49,212″N 8°12′17,017″E) and Isfjorden (62°34′36,844″N 7°42′5,0976″E). The areas have a marked mountainous topography that is characterised by large variations in elevation, with valleys and fjords. The local climate is characterised by relatively cool summers, mild winters, and high annual precipitation levels. The monthly temperatures for Isfjorden (meteorological station no 61350 [45]) range between 13.5°C in July and -1.3°C in January, whereas the mean temperature for Torjulvågen (meteorological station no 64510 [45]) ranges between 13.8°C in July and -0.5°C in January. The yearly precipitation in Isfjorden is on average 1211 mm, whereas Torjulvågen has a yearly average of 1160 mm. The mean temperature and precipitation have been calculated for the years 1961–1990 [45]. Our study area lies within the boreonemoral vegetation zone, and the forests are dominated by Scots pine (*Pinus sylvestris*), alder (*Alnus incana*), birch (*Betula* spp.), and scattered stands of Norway spruce (*Picea abies*) from extensive planting [46].

### Study design and data collection

A total of twelve transects were distributed in the study area: three at high elevation (approx. 400 m a.s.l) and three at low elevation (approx. 100 m a.s.l) in both localities. Twelve survey plots were placed along each transect, with randomised distances between 20 and 50 m [44,47]. The survey plots were examined for questing ticks with the cloth lure method [48] at approximately 14-day intervals from after the snow cover melted in spring to the first snow in autumn for the years 2011 (April 28th – November 23rd) and 2012 (April 26th – October 23rd). The specific cloth lure method used involved attaching a towel (50×100 cm) to the end of a rod as a flag [44,47]. Ticks were collected by dragging the cloth over the vegetation, and the towels were replaced with dry and clean towels when they became wet or dirty. Each survey plot covered a belt that was approximately 10 m long and 2 m wide (20 m^2^). The ticks were counted and removed from the towel after two drags on each side, and the total for each survey plot was registered. All flagging was performed during daytime hours. The cloth lure method only catches questing ticks, and this procedure typically underestimates the true abundance in the area [49] and may introduce a bias with regard to instar composition [33]. However, our goal in this study was to estimate temporal variation in questing densities rather than the true abundance, and the cloth lure method was a reasonable choice.

Temperature and RH were registered with locally installed weather loggers. A total of four loggers were used, one for each locality and elevation, and each logger covered three transects. The loggers were placed approximately one meter above ground to avoid extreme local conditions and variation at ground level. The weather data would thus be representative for all three transects covered, and still close to the tick questing height. The loggers registered the temperature and RH every 30 minutes, and VPD was calculated according to Gilbert’s method [36]. Time was registered at the beginning of each flagging in each survey plot and linked to the weather data from the closest weather logger.

### Statistical analyses

The overall aim of the analyses was firstly to describe the temporal pattern of tick questing at high and low elevation irrespective of mechanism. Such a temporal pattern of questing may be linked both to variation in both population densities and to questing activity. The questing activity part may further be subdivided into timing *per se* (life history strategy) and prevailing weather (conditions more or less favourable for questing). In the second step, we therefore aimed to separate the time (seasonal) component *per se* from the effect of prevailing weather. This step involves analysing tick densities as a response to prevailing weather variables (predictors), but also including time (date) as a predictor. Note that “hours of daylight” often used does not separate the same number of hours during fall and spring, and our approach allows for a different pattern in tick densities during spring and fall. In these two first analyses, we pooled adult ticks and nymphs, as adults were too few to warrant separate analysis. To make sure this did not impact our results, we also ran models with temporal trends as the predictor, but using proportion of nymphs of the total number of ticks counted as response variable.

We used the R statistical software (version 3.0.3) for all the analyses [50]. The main response variable is the number of ticks (adults and nymphs pooled) counted at the plot scale, i.e. a measure of density (ticks per 20 m^2^). Tick abundances are typically both zero-inflated and overdispersed relative to common approaches to count data, such as the Poisson distribution [51,52]. Initial analyses confirmed that a negative binomial probability distribution produced a better fit to our data than the Poisson distribution. The structure of the data with sampling along transects also warranted the use of mixed effect models (glmm), as data within a given transect are not independent of each other. When we analysed tick counts, we therefore used the negative binomial probability distribution in a mixed effect model setting with a random intercept term for transect identity that also accounted for zero inflation. This was performed with an integration of the AD model builder for R called glmmADMB (version 0.7.7) [53].

In the first descriptive analysis of seasonal trends, we analysed temporal patterns of questing tick densities with natural cubic spline function on the Julian date, using the “splines” library in R [50]. The reason for using splines is that these are very flexible and have no a priori assumption regarding the pattern of interest. We used the Akaike Information Criterion (AIC) for model selection [54]; for the selection of degrees of freedom in the spline function, we also partly used visual inspection of how the trend fitted the raw data. We analysed each year separately because the long break in the time series during winter would affect the spline function. A better model fit (lower AIC) with the inclusion of elevation categories (high/low) and the interaction between the spline function and elevation category, would indicate significant differences in the temporal pattern of questing densities between the two elevation categories. We also calculated mean values for each flagging session similarly for descriptive purposes. This was only done for low elevation due to limited sample size at high elevation.

The second analysis aimed to link the prevailing weather to questing tick density in the field, but we also aimed to determine if there were possible effects of time *per se*. Weather data from the climate loggers was linked to the tick data, using weather data from the 30-minute intervals at the beginning of each flagging. Flagging was performed 3906 times at the survey plot level, of which 3517 events had associated weather data. We only used data that could be linked to recordings from the climate loggers (N = 3517) in this procedure. Tick populations may also fluctuate between years due to unidentified factors like high winter mortality (2012 was on average 2.45°C warmer during Dec-Mar) or rodent cycles. We therefore included year in model selection to allow for different intercept between the years [55], but made sure to assess if it affected the estimates for prevailing weather that may also differ depending on elevation. A backward model selection procedure, including temperature, RH, VPD, year (as categorical), temperature squared, VPD squared, RH squared and vegetation height, was used to identify the best prevailing weather model [56]. The model had significant time trends in the residuals, indicative of an effect of time *per se* (as expected if timing of questing is also a life history trait). We therefore added as a covariate a spline function of the Julian date in interaction with the elevation category to the model. Further reduction of the covariates was then performed to produce the best model.

Some papers report temporal differences in questing between stages in the field [35] and also attachment patterns on hosts [57]. Adult ticks constitute ~10% of the total number of ticks in these coastal areas [44], and the number was suspected to be low for meaningful statistical inference. In the third analysis, we therefore checked for patterns in proportions of nymphs in the total count using several simple binomial regression analyses. We used the relative proportion between nymphs and adults as the response and the time, humidity measures, temperature and elevation as regressors. No significant relationships here would indicate that the adults and nymphs show a similar temporal pattern of questing densities in our area. A comparison between REML-based models, as described in [56], indicated that random effects were not necessary in this last analysis.

## Results

A total of 3078 ticks (2804 nymphs, 144 adult males and 130 adult females) were counted in all twelve transects. In the high-elevation transects, we recorded 86 nymphs, 6 adult males and 4 adult female ticks in 2011 and 56 nymphs, 7 adult males and 5 adult females in 2012. The recorded number for low elevations in 2011 was 1523 nymphs, 61 adult males and 61 adult females and 1139 nymphs, 70 adult males and 60 adult females in 2012.

Ticks were recorded over the entire sampling period for both years at low elevation (April 28th to November 23rd in 2011 and April 26th to October 23rd in 2012). At high elevation, the first appearance was June 6th in 2011, 36 days after flagging was initiated, and the last recorded tick was found on the last day of flagging (October 23rd). Ticks were recorded at high elevation on the same day as the first flagging in 2012 (May 23rd), and the last tick was recorded on October 10th, 13 days before flagging ceased. Therefore, ticks were found during almost the entire snow-free season.

### Description of seasonal trend

**Figure 1:**
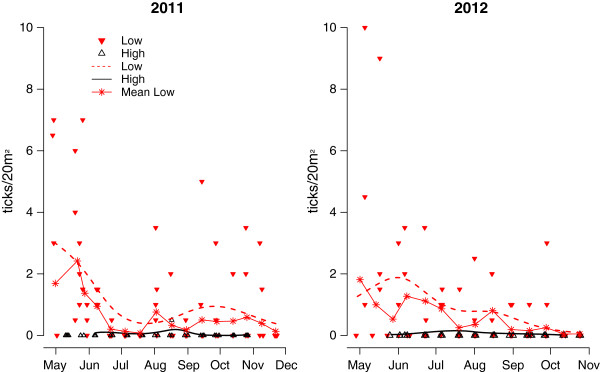
**Seasonal trends in questing tick densities at high and low elevations in 2011 and 2012 in Møre og Romsdal county, Norway.** The values on the y-axes are numbers of questing ticks collected per 20 m^2^ of flagging for each transect. The X-axes represent time. The trend is based on the best models from Table 1, and the intercept is the unadjusted estimate from the fixed effects. The red and black triangles show median questing tick density per transect for each bi-weekly flagging session. The red asterisks are predicted mean density of questing ticks for each bi-weekly flagging session (from a model with “transect” as random).

The interaction between elevation and date improved model fit for both 2011 and 2012, suggested differences in temporal pattern of questing tick densities between low and high elevations (Table 1, Figure 1). The questing season of ticks lasted much longer and tick densities were higher at low elevations, with a primary peak in May in both years and a smaller secondary peak in August-October in 2011, while the second peak in 2012 was not very clear. At high elevations, the questing season was much shorter and the density levels were overall much lower. The adult questing tick counts correlated with the questing nymph counts (Spearman’s rank correlation test, rho = 0.27, p <0.001). There was no trend in proportion of nymphs out of the total tick density by the visual inspection of the plots. Analyses revealed no relationship between proportion of nymphs and the temperature, elevation, RH, VPD, or no clear temporal pattern when “Juliandate” was entered as a spline with four degrees of freedom (p-values ranging between 0.96 and 0.40). Adults and nymphs are therefore pooled in the two first analyses.

**Table 1:** Model selection for the descriptive pattern of seasonal trend in questing tick density in 2011 and 2012

**Time**	**Elevation**	Elevation*Time	**AIC 2011**	**AIC 2012**
df = 1			3728	2834
df = 2			3723	2813
df = 3			3715	2814
df = 4			3718	2816
df = 5			3715	2817
df = 5	X		3687	2809
df = 4	X		3693	2808
df = 3	X		3690	2806
**df = 5**	X	X	**3614**	**2799**
df = 4	X	X	3640	2798
df = 3	X	X	NC	2796

All the models use the total tick abundance from each 20-m^2^ flagging as the response. Time as a natural cubic spline term, and elevation as a two level factor variable where high or low are predictors. df = degrees of freedom in the spline function; * = interaction term; NC = model did not converge. The chosen descriptive model is in bold, for 2012 it was chosen over the best model as visual inspection indicated a better fit.

### Estimating effects of prevailing weather

**Figure 2:**
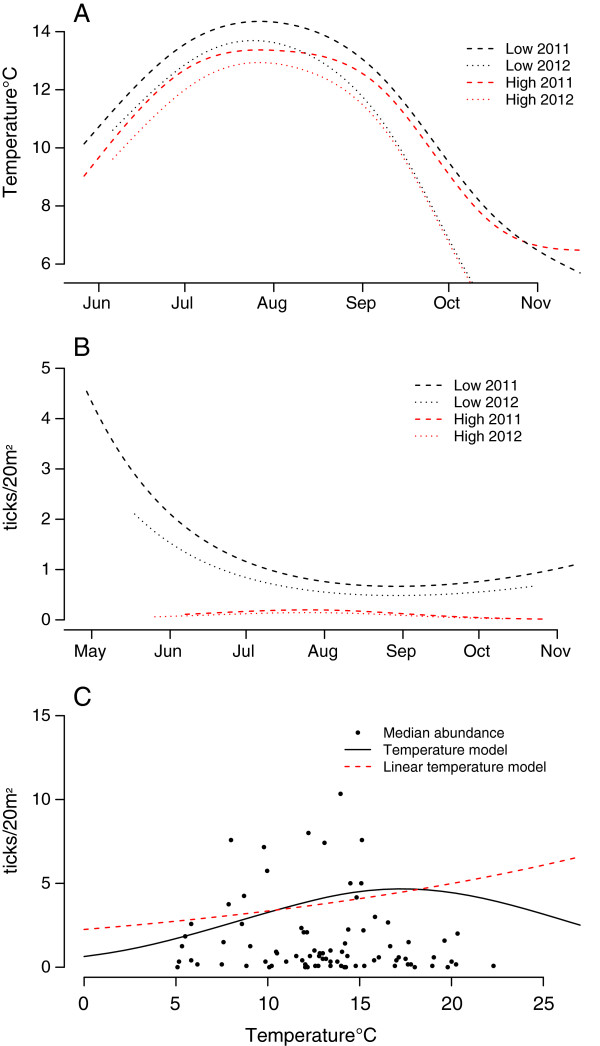
**Seasonal trend in temperature and a visualisation of the prevailing weather model. (A)** Seasonal trend in mean temperature fitted (5^th^ order spline) for low (~100 m a.s.l.) and high (~400 m a.s.l.) elevations in 2011 and 2012 in county Møre & Romsdal, Norway. **(B)** Seasonal trends in questing density of *Ixodes ricinus* ticks at high and low elevations in Møre & Romsdal county, Norway, for both years combined after controlling for the prevailing weather. The trend was based on the model from Tables 2 &3. Temperature was set to the estimated peak (17.3°C), and the intercept was the unadjusted estimate from the fixed effects. **(C)** Abundance of questing ticks per 20 m^2^ as an effect of temperature after controlling for the year and time trend effect. Points are median density of questing ticks per 20 m^2^ of flagging per transect/bi-weekly flagging session. The best model included a 2^nd^ order term for temperature, but a linear temperature effect is also included for comparison. The intercept is set to 2011, low elevation.

The best climate model included the year (categorical), temperature as a second-order polynomial, and a two-degree natural cubic spline of “Juliandate” with an elevation interaction (model selection procedure is shown in Table 2 and parameter estimates in Table 3). There was considerable variation in temperature both within and between elevations and years (Figure 2A), and the inclusion of elevation and year (as factor) had limited impact on the estimated effect of temperature (Table 4). The time trend still showed a bimodal tendency at low elevations, even when controlling for the prevailing weather (Figure 2B). Questing tick densities were considerably lower at low temperatures. Only three ticks were found at temperatures lower than 5°C. The density of questing ticks increased exponentially and levelled off above 15-17°C. Although not a sharp peak, questing tick densities appeared to decrease with temperature increases above 15-17°C (Figure 2C). This finding indicates that the time trend and temperature are not entirely independent, such that the high tick densities in spring are partly an effect of date, whereas warmer temperatures later in the summer are linked to lower questing tick densities.

**Table 2:** Model selection procedure with the climate variables and year as predictors and the total questing tick density as the response

**Year**	**Relative humidity (RH)**	**Vapour pressure deficit (VPD)**	**Temperature**	**Vegetation height**	**(RH/VPD)**^**2**^	**(Temperature)**^**2**^	(RH/VPD)* Temperature	(RH/VPD)* (Temperature)^**2**^	**(RH/VPD)**^2^* Temperature	**(RH/VPD)**^2^* (Temperature)^**2**^	**Elevation category**	**date**	**ns (date, df = 2)**	Elevation category*date	**AIC**	**∆ AIC**
X	X		X	X	X	X	X	X	X	X					5966.4	127.8
X		X	X	X	X	X	X	X	X	X					5971.3	132.7
X		X	X		X	X	X	X	X	X					5965.4	126.8
X	X		X	X	X	X	X	X	X						5964.5	125.9
X	X		X	X	X	X	X	X		X					5964.5	125.9
X	X		X	X	X	X	X		X	X					5964.5	125.9
X	X		X	X	X	X		X	X	X					5964.7	126.1
X	X		X		X	X	X	X	X						5963.4	124.8
X	X		X		X	X	X	X		X					5964.5	125.9
X	X		X		X	X	X		X	X					5963.5	124.9
X	X		X		X	X		X	X	X					5963.7	125.1
X	X		X		X	X	X	X							5965.2	126.6
X	X		X		X	X	X		X						5969.0	130.4
X	X		X		X	X		X	X						5967.0	128.4
X	X		X		X	X	X								5967.0	128.4
X	X		X		X	X		X							5966.1	127.5
X	X		X		X	X									5965.5	126.9
X	X		X		X										5983.5	144.9
X	X		X			X									5964.9	126.3
X	X		X												5980.9	142.3
X	X														5983.5	144.9
X			X			X									5993.6	155
	X		X			X									5988.9	150.3
X	X		X			X					X		X	X	5840.3	1.7
X	X		X			X					X	X		X	5882.7	44.1
X	X		X			X					X		X		5874.3	35.7
X	X		X			X					X	X			5880.9	42.3
X	X		X								X		X	X	5848.1	9.5
**X**			**X**			**X**					**X**		**X**	**X**	**5838.6**	0
	X		X			X					X		X	X	5848.1	9.5
X	X										X		X	X	5849.4	10.8

X = term included; AIC = Akaike’s information criterion; ∆ AIC = the difference in AIC value between the given model and the model with the lowest AIC; * = interaction term, and all other variables are additive. Note that in the interactions, the term (RH/VPD) means either relative humidity or vapour pressure deficit, depending on which variable was included as the linear effect.

Bold face indicates the best prevailing weather model.

**Table 3:** The output (parameter estimates, standard errors, z- and p-values) of the model explaining the questing tick density as an effect of prevailing weather

**Coefficients**				
	**Estimate**	**Std. error**	**Z value**	**Pr(<|z|)**
Intercept	-5.2	0.69	-7.54	<0.001
Year 2012	-0.32	0.082	-3.92	<0.001
Temperature	0.23	0.058	3.98	<0.001
(Temperature)^2^	-0.0066	0.0020	-3.37	<0.001
Low elevation	4.8	0.68	7.01	<0.001
ns(date, df =2) 1	1.6	0.74	2.16	0.030
ns(date, df =2) 2	-3.0	0.52	-5.78	<0.001
Low elevation* ns(date, df = 2) 1	-5.0	0.83	-6.07	<0.001
Low elevation* ns(date, df = 2) 2	2.7	0.57	4.79	<0.001

The term “*” means interaction, and “ns” is natural cubic spline; “df” specifies the degrees of freedom used in the spline. The baseline for the model is year 2011 and “high” elevation.

**Table 4:** An overview of different models to ensure consistency in parameter estimates of temperature effects

**Year**	**Temp**	**(Temp)**^**2**^	**Elevation (high vs. low)**	**ns(Date, df = 2)**	Elevation* ns(Date, df = 2)	**Estimate Temp**	**Estimate (Temp)**^**2**^	**AIC**	**Resid trend**
**X**	**X**	**X**	**X**	**X**	**X**	**0.23**	**0.0066**	**5838.6**	**No**
X	X	X	X	X		0.22	0.0062	5872.42	No
X	X	X		X		0.22	0.0062	5882.58	No
X	X	X	X			0.31	0.0092	5893.2	Yes
X	X	X				0.31	0.009	5593.62	Yes
X	X		X	X	X	0.040		5847.86	No
X	X		X	X		0.049		5881.1	No
X	X			X		0.050		5891.34	No
X	X		X			0.056		6003.92	Yes
X	X					0.056		6014.36	Yes
X	X	X	X	X	X	0.24	0.0069	5847.18	No
	X	X	X	X		0.22	0.0062	5872.42	No
X	X	X		X		0.24	0.0068	5893.62	No
	X	X	X			0.31	0.0094	5996.04	Yes
	X	X				0.31	0.0094	6006.56	Yes
	X		X	X	X	0.048		5857.68	No
X	X		X	X		0.048		5894.48	No
X	X			X		0.048		5904.76	No
	X		X			0.049		6017.82	Yes
	X					0.049		6028.36	Yes

Inclusion of a 2^nd^ degree term for temperature improves the AIC-value. ns = natural cubic spline, * = interaction, resid trend = “Yes” means that there is a time trend in the residuals. X = term included in the model. Bold face indicates the chosen prevailing weather model. Note that this model is the same as the best model from Table 2 with parameter estimates presented in Table 3.

## Discussion

Ticks and their associated diseases are regarded as highly sensitive to climate change in Europe. It is therefore relevant to determine how the temporal pattern of the tick vector is influenced by prevailing weather conditions under different climate regimes at low and high elevations. We present evidence that the seasonal pattern of questing tick densities differed depending on elevation in an area close to the northern latitudinal limit in Europe (Figure 1). A long period of seasonal questing and high overall densities with a marked spring peak and, at least one year, a weaker fall peak was recorded at low elevation, whereas at high elevation, the questing season was considerably shorter with lower overall densities and no clear peaks. As expected, tick questing density was strongly hampered by low temperatures early in the season. Only three ticks were found at temperatures <5°C, with an increase in questing tick densities toward 15-17°C and then a decline in densities as the temperature increased towards summer (Figure 2C).

### Onset and peak in seasonal tick questing densities

The temporal pattern of questing tick densities reflects a composite of tick population densities in the litter and the questing activity of these. Various patterns in the phenology of *I. ricinus* are observed throughout its wide distribution range, and much of this variation is likely linked to the climate regime [29,38,58]. Reported patterns vary from unimodal, with either a summer (Europe and North Africa) or a winter peak (North Africa), to bimodal patterns with varying peak tick questing densities [34,35]. In Ireland and Switzerland, bimodal nymphal patterns with a major spring peak and a smaller autumn peak have been observed [59,60], whereas ticks from British Isles and Italy show differing patterns of questing, from a single mid- or late-summer peak to a bimodal pattern with both spring and autumn maxima [29,38,59]. Our study provides evidence of differing temporal variation in questing tick densities based on elevation, even within the same region. We found a marked spring peak and at least in one year a weaker fall peak in the temporal pattern of tick questing densities at low elevations (~100 m a.s.l.) and a pattern of overall much lower tick densities at higher elevations (~400 m a.s.l.). Studies of tick seasonality in Swedish lowland show bimodal patterns in questing densities, with questing peaks in May and August/September [34,61]. The midsummer depression was discussed in relation to dryer conditions during this period, as drought will force ticks to move down to the litter layer to rehydrate [28,29]. The Atlantic climate on the western Norwegian coast in our study is unlikely to cause desiccation stress in midsummer, in contrast to on the eastern Swedish coastline. We found no evidence for a reduction in tick questing densities with drier conditions, and the midsummer depression in our data is likely due to other mechanisms than midsummer drought.

Although low tick densities at high elevations have been reported previously [36,44], it does not appear to be a universal feature. On north-facing slopes in the Swiss Alps, the abundance of questing nymphs was highest at the most elevated localities [35,62]. These areas have a warm and dry climate, but elevated north-facing localities may have a more favourable microclimate for ticks in continental Europe. The ticks in lowland areas close to sea level in our coastal area of Norway started questing at approximately the end of April, whereas the first ticks at ~400 m a.s.l. appeared in May or June and with a fairly short questing season. The onset of nymphal and adult tick questing started as early as March in the Swiss Alps [35]. The onset of nymphal and adult tick questing in England often starts in February, but ticks can sometimes be active year-round in the south of England where winter temperatures are significantly higher than in our study area [29].

We found that temperature explained a considerable portion of the tick questing densities during summer at both high and low elevations. A residual time trend was still present with a reduction toward August (Figure 2B) and then a slight increase during autumn. A comparison of the seasonal trend (Figure 1) and the trend after controlling for weather conditions (Figure 2B) show that it is mainly the onset and the end of the questing season that are temperature driven. The midsummer low is likely due to other mechanisms that are not recorded in the present study. This may be driven by life cycle history such as timing of egg laying, hatching of the different tick stages, diapause induction and mortality. The number of questing ticks is also affected by proportion of the population being on a host [43]. The high recruitment rate in spring at low elevations indicates that many newly emerged ticks are reactivated after behavioural quiescence or are activated for the first time [29,58]. Ticks will then get picked up by hosts or die towards summer, and this will in turn lead to reduced numbers that are questing as the temperature increases. Such a process may lead to higher densities of questing ticks at temperatures lower than the optimal for tick questing activity (Figure 2C). A second peak may be the result of high summer temperatures and a long season. Interstadial periods of *Ixodes* species can be prolonged (i.e. adjusted) by a variable delay in the onset of diapause. Photoperiod has been identified as a major cue for entering into the diapause. The date of diapause onset shows a latitudinal gradient and may act together with other factors such as decreasing temperature and increasing tick age [43]. Theoretically, ticks (e.g., larvae) can feed in spring, moult during summer and then start questing (e.g., as nymphs) again in autumn of the same year [63]. However, experimental evidence from Germany indicates that nymphs moulting from larvae do not start questing the same year (O. Kahl, pers. comm.). A study on *I. scapularis* by Lindsay *et al*. [64] also suggests this. Decreasing temperatures with elevation will lead to shorter periods of temperatures that allow for tick activity, possibly further extending the tick life cycle. It has also been suggested that red deer migration in spring may create a vehicle for ticks [44]. It is possible that the small tick populations at high elevations are dependent on deer migration to sustain a stable population, i.e., a source-sink system, though this remains to be documented.

### Temperature, day length and onset of questing activity

Tick questing started as expected at approximately 5°C, and no nymphs were captured below 3.5°C. There was a marked increase in questing tick densities toward 15-17°C. We recorded no difference between adults and nymphs in the temporal pattern, and the first adult tick was collected at 4.5°C. Our measurements were taken ~1 m above the ground to ensure it to be representative for all transects covered by each logger. The microclimate may nevertheless have been warmer or colder at the exact location of the active tick, since we only had one logger per 3 transects and not at each exact spot used for flagging. Other studies report that onset appeared to be determined by temperatures ranging above 7°C in the north of Great Britain if day length was not limiting for tick activity [29,36] or 7°C - 8.5°C in Switzerland [25,35]. *I. ricinus* is suggested to respond to changes in day length [65]. The onset of tick activity is suggested to be affected by both the temperature threshold and changes in day length. Recently, it was also shown that ticks differed in the likelihood of questing at a given temperature depending on geographical origin, suggesting adaptations to the local climatic conditions [27].

Tick questing densities decreased significantly when the temperatures rose above 15-17°C. However, ticks have been shown to be active and abundant at much higher temperatures elsewhere, for instance, in southern England [29]. The reduction in tick densities with increasing temperature found in our study might theoretically be related to the RH or VPD becoming limiting at higher temperatures. One controlled laboratory study that tested *I. ricinus* activity at 25°C and 60% RH, 25°C and 85% RH and 15°C and 85% RH suggested that the optimal condition for questing was closer to 15°C under the target RH, which corresponded to an optimal VPD of 1.9 mmHg [65]. Therefore, it is difficult to conclude whether it was the temperature or desiccation stress that was the most important factor contributing to questing activity in those experiments. Both negative and positive correlations with temperature have been found in other studies. A negative correlation with temperature in a more continental climate in Sweden [66] compared to a positive correlation in an Atlantic climate in Ireland [59] suggest that differences in temperature response may be related to climate. High temperatures may induce desiccation stress in dry/continental areas.

However, in our study, the seasonal trend after controlling for climatic conditions still show a midsummer reduction in questing densities (Figure 2B), indicating that the temperature correlation was confounded with date effects. Under warm and dry conditions, ticks that successfully feed during spring spend midsummer in developmental quiescence, which leads to reduced density during the warmest season. Our study in a humid climate indicates that such a mid-summer drop in questing tick densities likely may also have other causes.

### Tick questing densities, climate and beyond

Understanding the temporal variation of questing tick densities requires disentangling the interplay of several other aspects than climate. Between year variation and differences between elevations is clearly partly due to climate. However, the estimate for the temperature effect was robust to the inclusion of both year (as factor) and elevation (as factor). This suggests that low questing tick densities at cold temperatures is independent of elevation or annual fluctuations, and that the length of questing season is mainly temperature dependent. Temperature varied to the extent that temperatures at high elevations in 2011 were comparable to low elevation in 2012 (Figure 2A). This could be the reason behind the earlier cease in questing at both elevations, a later spring peak and a less pronounced bimodality in 2012. The inclusion of a time trend in interaction with elevation was necessary to provide a model without a residual time trend (Table 4). Clearly, patterns in questing linked to time (seasonality) *per se* are part of a tick’s life history traits. This could also be modelled with “hours of daylight”, however, the same hour of daylight may link differently to tick questing density in spring and fall, and we therefore prefer to model this effect as a date variable. Furthermore, the tick population is not stable in a given season. New individuals molting are entering the questing tick population, while there is also a depletion of the questing tick population linked both to mortality and those succeeding in finding a host. In Europe, dense red deer and roe deer populations are regarded as important to maintain high densities of ticks. The adult tick female requires a large blood meal before reproduction [28], and the most abundant large hosts in Scandinavia are cervids. There is strong evidence that increasing tick abundance in Scandinavia follow increasing red deer and roe deer populations [16,18,67]. How these dense deer populations may affect the timing of questing tick densities is a yet unresolved question, but it may cause a more rapid depletion of the questing tick population towards summer as ticks are more likely to find a host. In addition, although climate certainly varies depending on elevation, the host community composition and density may also differ to some extent. Currently, there is not sufficient data to separate all of the different processes that might contribute to the seasonal pattern of questing tick densities.

## Conclusions

We have shown that local differences in questing tick densities may depend on climatic conditions. In the present study, we have linked this to variation in elevation. We found no evidence of desiccation stress in our humid study site, but low temperatures significantly lowered questing tick densities especially early and late in the season, and thus seemed to limit the duration of the questing season. Therefore, it is likely that climate warming will lead to a longer questing season, higher overall densities and a marked spring peak and a weaker fall peak in questing tick densities (similar to the conditions at low elevations) in regions currently showing a short questing season and lower overall questing tick densities (similar to the conditions at high elevations). The likely future scenario of climate change on these systems is therefore increased tick densities and distribution range. This also includes the various pathogens that follow these disease-transmitting vectors.

## Competing interests

The authors affirm that they have no competing interests.

## Authors’ contributions

AM, LG, LQ and IK designed the study. LQ, HV, and AM designed and carried out data analysis. LQ and AM drafted the manuscript. All authors read and approved the final version of the manuscript.

## References

[B1] Walther G-R Post E Convey P Menzel A Parmesan C Beebee TJC Fromentin J-M Hoegh-Guldberg O Bairlein F Ecological responses to recent climate change Nature 2002 416 389 395 10.1038/416389a 11919621

[B2] Parmesan C Yohe G A globally coherent fingerprint of climate change impacts across natural systems Nature 2003 421 37 42 10.1038/nature01286 12511946

[B3] Hamann A Wang T Potential effects of climate change on ecosystem and tree species distribution in British Coloumbia Ecology 2006 87 2773 2786 10.1890/0012-9658(2006)87[2773:PEOCCO]2.0.CO;2 17168022

[B4] Lafferty KD The ecology of climate change and infectious diseases Ecology 2009 90 888 900 10.1890/08-0079.1 19449681

[B5] Sutherst RW Floyd RB Bourne AS Dallwitz MJ Cattle grazing behavior regulates tick populations Experientia 1986 42 194 196 10.1007/BF01952465

[B6] Zhang Y Bi P Hiller JE Climate change and the transmission of vector-borne diseases: a review Asia Pac J Public Health 2008 20 64 76 10.1177/1010539507308385 19124300

[B7] Rejmánková E Grieco J Achee N Masuoka P Pope K Roberts D Higashi RM Collinge SK, Ray C Freshwater community interactions and malaria Dis Ecol 2006 USA: Oxford University Press 90 104

[B8] Ostfeld RS Canham CD Oggenfuss K Winchcombe RJ Keesing F Climate, deer, rodents, and acorns as determinants of variation in Lyme-disease risk PLoS Biol 2006 4 e145 10.1371/journal.pbio.0040145 16669698 PMC1457019

[B9] Randolph SE Green RM Peacey MF Rogers DJ Seasonal synchrony: the key to tick-borne encephalitis foci identified by satellite data Parasitology 2000 121 15 23 10.1017/S0031182099006083 11085221

[B10] Randolph SE Tick-borne disease systems emerge from the shadows: the beauty lies in molecular detail, the message in epidemiology Parasitology 2009 136 1403 1413 10.1017/S0031182009005782 19366480

[B11] Jaenson TGT Lindgren E The range of *Ixodes ricinus* and the risk of contracting Lyme borreliosis will increase northwards when the vegetation period becomes longer Ticks Tick Borne Dis 2011 2 44 49 10.1016/j.ttbdis.2010.10.006 21771536

[B12] Woldehiwet Z Immune evasion and immunosuppression by *Anaplasma phagocytophilum*, the causative agent of tick-borne fever of ruminants and human granulocytic anaplasmosis Vet J 2008 175 37 44 10.1016/j.tvjl.2006.11.019 17275372

[B13] Woldehiwet Z *Anaplasma phagocytophilum* in ruminants in Europe Ann N Y Acad Sci 2006 1078 446 460 10.1196/annals.1374.084 17114753

[B14] Stuen S *Anaplasma phagocytophilum* - the most widespread tick-borne infection in animals in Europe Vet Res Commun 2007 31 Suppl 1 79 84 17682851 10.1007/s11259-007-0071-y

[B15] Hasle G Leinaas HP Røed KH Øines Ø Transport of *Babesia venatorum*-infected *Ixodes ricinus* to Norway by northward migrating passerine birds Acta Vet Scand 2011 53 41 10.1186/1751-0147-53-41 21699719 PMC3132728

[B16] Jaenson TGT Jaenson DGE Eisen L Petersson E Lindgren E Changes in the geographical distribution and abundance of the *tick Ixodes ricinus* during the past 30 years in Sweden Parasit Vectors 2012 5 8 10.1186/1756-3305-5-8 22233771 PMC3311093

[B17] Jore S Viljugrein H Hofshagen M Brun-Hansen H Kristoffersen AB Nygard K Brun E Ottesen P Saevik BK Ytrehus B Multi-source analysis reveals latitudinal and altitudinal shifts in range of *Ixodes ricinus* at its northern distribution limit Parasit Vectors 2011 4 84 10.1186/1756-3305-4-84 21595949 PMC3123645

[B18] Jensen PM Jespersen JB Five decades of tick–man interaction in Denmark – an analysis Exp Appl Acarol 2005 35 131 146 10.1007/s10493-004-1991-7 15777006

[B19] Kirby AD Smith AA Benton TG Hudson PJ Rising burden of immature sheep ticks (*Ixodes ricinus*) on red grouse (*Lagopus lagopus scoticus*) chicks in the Scottish uplands Med Vet Entomol 2004 18 67 70 10.1111/j.0269-283X.2004.0479.x 15009449

[B20] Materna J Daniel M Danielová V Altitudinal distribution limit of the tick *Ixodes ricinus* shifted considerably towards higher altitudes in central Europe: results of three years monitoring in the Krkonose Mts. (Czech Republic) Cent Eur J Public Health 2005 13 24 28 15859176

[B21] Materna J Daniel M Metelka L Harčarik J The vertical distribution, density and the development of the tick *Ixodes ricinus* in mountain areas influenced by climate changes (The Krkonoše Mts., Czech Republic) Int J Med Microbiol 2008 298 25 37

[B22] Dobson ADM Randolph SE Modelling the effects of recent changes in climate, host density and acaricide treatments on population dynamics of *Ixodes ricinus* in the UK J Appl Ecol 2011 48 1029 1037 10.1111/j.1365-2664.2011.02004.x

[B23] Medlock JM Hansford KM Bormane A Derdakova M Estrada-Peña A George J-C Golovljova I Jaenson TGT Jensen J-K Jensen PM Kazimirova M Oteo JA Papa A Pfister K Plantard O Randolph SE Rizzoli A Santos-Silva MM Sprong H Vial L Hendrickx G Zeller H Van Bortel W Driving forces for changes in geographical distribution of *Ixodes ricinus* ticks in Europe Parasit Vectors 2013 6 1 10.1186/1756-3305-6-1 23281838 PMC3549795

[B24] Randolph S Chemini C Furlanello C Genchi C Hails R Hudson PJ Jones LD Medley G Norman R Rizzoli A Smith G Woolhouse M Rizzoli AP Grenfell B Heesterbeek H Dobson A The ecology of tick-borne infections in wildlife reservoirs Ecol Wildl Dis 2002 1 Oxford, United Kingdom: Oxford University Press 119 138

[B25] Perret J-L Guigoz E Rais O Gern L Influence of saturation deficit and temperature on *Ixodes ricinus* tick questing activity in a Lyme borreliosis-endemic area (Switzerland) Parasitol Res 2000 86 554 557 10.1007/s004360000209 10935905

[B26] Dobson ADM Finnie TJR Randolph SE A modified matrix model to describe the seasonal population ecology of the European tick *Ixodes ricinus* J Appl Ecol 2011 48 1017 1028 10.1111/j.1365-2664.2011.02003.x

[B27] Gilbert L Aungier J Tomkins J Climate of origin affects tick (*Ixodes ricinus*) host-seeking behaviour in response to temperature: implications for resilience to climate change? Ecol Evol 2014 in press 10.1002/ece3.1014 PMC3997332 24772293

[B28] Sonenshine DE Roe RM Biology of Ticks Volume 1 2014 2 New York: Oxford University Press, USA 560

[B29] Randolph SE Tick ecology: processes and patterns behind the epidemiological risk posed by ixodid ticks as vectors Parasitology 2004 129 Suppl S37 S65 15938504 10.1017/s0031182004004925

[B30] Van Gent M Assessing behavioral aspects of Ixodes ricinus in relation to infection with Borrelia burgdorferi s.l PhD thesis 2009 Wageningen University, Laboratory of Entomology

[B31] Randolph SE Storey K Impact of microclimate on immature tick-rodent host interactions (Acari: Ixodidae): implications for parasite transmission J Med Entomol 1999 36 741 748 10593075 10.1093/jmedent/36.6.741

[B32] Lees AD Milne A The seasonal and diurnal activities of individual sheep ticks (*Ixodes ricinus* L.) Parasitology 1951 41 189 10.1017/S0031182000084031 14911213

[B33] Mejlon HA Jaenson TGT Questing behaviour of *Ixodes ricinus* ticks (Acari: Ixodidae) Exp Appl Acarol 1997 21 747 754 10.1023/A:1018421105231

[B34] Mejlon HA Jaenson TGT Seasonal prevalence of *Borrelia burgdorferi* in *Ixodes ricinus* in different vegetation types in Sweden Scand J Infect Dis 1993 25 449 456 10.3109/00365549309008526 8248744

[B35] Cadenas FM Rais O Jouda F Douet V Humair P Moret J Gern L Phenology of *Ixodes ricinus* and Infection with *Borrelia burgdorferi* sensu lato along a north- and south-facing altitudinal gradient on Chaumont Mountain, Switzerland J Med Entomol 2007 44 683 693 10.1603/0022-2585(2007)44[683:POIRAI]2.0.CO;2 17695026

[B36] Gilbert L Altitudinal patterns of tick and host abundance: a potential role for climate change in regulating tick-borne diseases? Oecologia 2010 162 217 225 10.1007/s00442-009-1430-x 19685082

[B37] Knap N Durmiši E Saksida A Korva M Petrovec M Avšič-Županc T Influence of climatic factors on dynamics of questing *Ixodes ricinus* ticks in Slovenia Vet Parasitol 2009 164 275 281 10.1016/j.vetpar.2009.06.001 19560275

[B38] Tagliapietra V Rosà R Arnoldi D Cagnacci F Capelli G Montarsi F Hauffe H Rizzoli A Saturation deficit and deer density affect questing activity and local abundance of *Ixodes ricinus* (Acari, Ixodidae) in Italy Vet Parasitol 2011 183 114 124 10.1016/j.vetpar.2011.07.022 21820245

[B39] Egyed L Élő P Sréter-Lancz Z Széll Z Balogh Z Sréter T Seasonal activity and tick-borne pathogen infection rates of *Ixodes ricinus* ticks in Hungary Ticks Tick Borne Dis 2012 3 90 94 10.1016/j.ttbdis.2012.01.002 22445929

[B40] Li S Heyman P Cochez C Simons L Vanwambeke SO A multi-level analysis of the relationship between environmental factors and questing *Ixodes ricinus* dynamics in Belgium Parasit Vectors 2012 5 149 10.1186/1756-3305-5-149 22830528 PMC3419667

[B41] Randolph SE The shifting landscape of tick-borne zoonoses: tick-borne encephalitis and Lyme borreliosis in Europe Philos Trans R Soc London Ser B Biol Sci 2001 356 1045 1056 10.1098/rstb.2001.0893 11516382 PMC1088499

[B42] Ogden NH Bigras-Poulin M Hanincova K Maarouf A O’callaghan CJ Kurtenbach K Projected effects of climate change on tick phenology and fitness of pathogens transmitted by the North American tick *Ixodes scapularis* J Theor Biol 2008 254 621 632 10.1016/j.jtbi.2008.06.020 18634803

[B43] Randolph SE Sonenshine DE, Roe RM Ecology of non-nidicolous ticks Biol ticks, Vol 2 2014 2 New York: Oxford University Press 3 60

[B44] Qviller L Risnes-Olsen N Bærum KM Meisingset EL Loe LE Ytrehus B Viljugrein H Mysterud A Landscape level variation in tick abundance relative to seasonal migration in red deer PLoS One 2013 8 10 10.1371/journal.pone.0071299 PMC3739797 23951125

[B45] eKlima http://sharki.oslo.dnmi.no/portal/page?_pageid=73,39035,73_39080&_dad=portal&_schema=PORTAL

[B46] Mysterud A Langvatn R Yoccoz NG Stenseth NC Large-scale habitat variability, delayed density effects and red deer populations in Norway J Anim Ecol 2002 71 569 580 10.1046/j.1365-2656.2002.00622.x

[B47] Steigedal HH Loe LE Grøva L Mysterud A The effect of sheep (*Ovis aries*) presence on the abundance of ticks (*Ixodes ricinus*) Acta Agric Scand Sect A - Anim Sci 2013 63 111 120

[B48] Vassallo M Pichon B Cabaret J Figureau C Perez-Eid C Methodology for sampling questing nymphs of *Ixodes ricinus* (Acari: Ixodidae), the principal vector of Lyme disease in Europe J Med Entomol 2000 37 335 339 10.1603/0022-2585(2000)037[0335:MFSQNO]2.0.CO;2 15535574

[B49] Dobson ADM Taylor JL Randolph SE Tick (*Ixodes ricinus*) abundance and seasonality at recreational sites in the UK: Hazards in relation to fine-scale habitat types revealed by complementary sampling methods Ticks Tick Borne Dis 2011 2 67 74 10.1016/j.ttbdis.2011.03.002 21771540

[B50] R: A Language and Environment for Statistical Computing http://www.R-project.org/

[B51] Zuur AF Ieno EN Walker N Saveliev AA Smith GM Mixed Effects Models and Extensions in Ecology with R 2009 New York: Springer 596

[B52] James MC Bowman AS Forbes KJ Lewis F McLeod JE Gilbert L Environmental determinants of *Ixodes ricinus* ticks and the incidence of *Borrelia burgdorferi* sensu lato, the agent of Lyme borreliosis, in Scotland Parasitology 2013 140 237 246 10.1017/S003118201200145X 23036286

[B53] glmmADMB: Generalized Linear Mixed Models Using Ad Model Builderhttp://glmmadmb.r-forge.r-project.org/

[B54] Burnham KP Anderson DR Model Selection and Multimodel Inference: A Practical Information-Theoretic Approach 2002 2 New York: Springer

[B55] Zuur AF Ieno EN Walker N Saveliev AA Smith GM Limitations of linear regression applied on ecological data Mix Eff Model Extensions Ecol with R 2009 New York: Springer 11 33

[B56] Zuur AF Ieno EN Walker N Saveliev AA Smith GM Mixed effects modelling for nested data Mix Eff Model Extensions Ecol with R 2009 New York: Springer 101 143

[B57] Handeland K Qviller L Vikøren T Viljugrein H Lillehaug A Davidson RK *Ixodes ricinus* infestation in free-ranging cervids in Norway–a study based upon ear examinations of hunted animals Vet Parasitol 2013 195 142 149 10.1016/j.vetpar.2013.02.012 23541678

[B58] Randolph SE Green RM Hoodless AN Peacey MF An empirical quantitative framework for the seasonal population dynamics of the tick *Ixodes ricinus* Int J Parasitol 2002 32 979 989 10.1016/S0020-7519(02)00030-9 12076627

[B59] Gray JS Studies on the dynamics of active populations of the sheep tick, *Ixodes ricinus* L. in Co. Wicklow, Ireland Acarologia 1984 25 167 178 6485729

[B60] Jouda F Perret J-L Gern L *Ixodes ricinus* density, and distribution and prevalence of *Borrelia burgdorferi* Sensu Lato infection along an altitudinal gradient J Med Entomol 2004 41 162 169 10.1603/0022-2585-41.2.162 15061274

[B61] Nilsson A Seasonal occurrence of *Ixodes ricinus* (Acari) in vegetation and on small mammals in Southern Sweden Holarct Ecol 1988 11 161 165

[B62] Burri C Moran Cadenas F Douet V Moret J Gern L *Ixodes ricinus* density and infection prevalence of *Borrelia burgdorferi* sensu lato along a north-facing altitudinal gradient in the Rhône Valley (Switzerland) Vector Borne Zoonotic Dis 2007 7 50 58 10.1089/vbz.2006.0569 17417957

[B63] Gray JS The fecundity of *ixodes ricinus* (L.) (Acarina: Ixodidae) and the mortality of its developmental stages under field conditions Bull Entomol Res 1981 71 533 10.1017/S0007485300008543

[B64] Lindsay LR Barker IK Surgeoner GA McEwen SA Gillespie TJ Addison EM Survival and development of the different life stages of *Ixodes scapularis* (Acari: Ixodidae) held within four habitats on Long Point, Ontario, Canada J Med Entomol 1998 35 189 199 9615533 10.1093/jmedent/35.3.189

[B65] Perret J-L Guerin PM Diehl PA Vlimant M Gern L Darkness induces mobility, and saturation deficit limits questing duration, in the tick *Ixodes ricinus* J Exp Biol 2003 206 1809 1815 10.1242/jeb.00345 12728002

[B66] Mejlon HA Diel activity of *Ixodes ricinus* Acari: Ixodidae at two locations near Stockholm, Sweden Exp Appl Acarol 1997 21 247 256 10.1023/A:1018446921644 9178511

[B67] Jore S Vanwambeke SO Viljugrein H Isaksen K Kristoffersen AB Woldehiwet Z Johansen B Brun E Brun-Hansen H Westermann S Larsen I-L Ytrehus B Hofshagen M Climate and environmental change drives *Ixodes ricinus* geographical expansion at the northern range margin Parasit Vectors 2014 7 11 10.1186/1756-3305-7-11 24401487 PMC3895670

